# Emergency department point-of-care ultrasound for upper extremity deep venous thrombosis ED POCUS for upper extremity DVT

**DOI:** 10.1186/s12245-021-00391-4

**Published:** 2021-11-04

**Authors:** Gregory Tan, Mingwei Ng

**Affiliations:** Department of Emergency Medicine, Singapore General Hospital, Outram Rd, Singapore, 169608 Singapore

**Keywords:** Upper extremity Deep vein thrombosis, Ultrasound, Augmentation, Colour Doppler

## Abstract

**Background:**

Upper extremity deep vein thrombosis (UEDVT) is an uncommon disease but has to be carefully considered in patients with isolated unilateral upper limb swelling due to its potential to cause devastating complications and sequelae such as pulmonary embolism and septic thrombophlebitis. Given the extreme rarity of this condition, it is not surprising that point-of-care ultrasonographic evaluation of the upper limb for deep venous thrombosis is hardly ever performed in the emergency department. This case report serves to highlight how point-of-care ultrasonographic evaluation of the upper extremity venous system could be incorporated as a tool in the diagnostic armamentarium of the emergency physician.

**Case presentation:**

A 51-year-old Chinese gentleman presented to the emergency department with a 1-day duration of progressive right upper extremity swelling and pain. On examination, his hemodynamic parameters were stable with no tachycardia. He was noted to have a hyperaemic and grossly swollen but non-tender right upper limb. Distal pulses remained strong. Point-of-care ultrasonography of his right upper limb venous system with Doppler colour flow and single-point augmentation with the arm squeeze manoeuvre immediately confirmed the diagnosis of right upper extremity deep venous thrombosis, which in turn permitted anticoagulation to be instituted promptly whilst in the emergency department.

**Conclusion:**

The use of point-of-care ultrasonography of the upper limb venous system can prove invaluable as a rapid, non-invasive technique to facilitate expedient diagnosis of and early intervention for UEDVT in the emergency department.

## Background

Upper extremity deep vein thrombosis (UEDVT) represents only 1–4% of all DVTs [1, 2] but is associated with similarly devastating complications such as pulmonary embolism, post-thrombotic syndrome, and septic thrombophlebitis [2]. Due to the exceeding rarity, there is scant literature and evidence on the optimal diagnostic approach to patients with suspected UEDVT in the emergency department. Diagnosis is therefore often either missed or delayed till formal scans by radiology can be obtained. Whilst Doppler ultrasound has long been utilized as a bedside technique to detect venous obstruction in the lower extremities [3], bedside point-of-care ultrasound (POCUS) has hardly been applied for evaluation of the upper extremity venous system in the emergency department and could prove invaluable for early diagnosis and management of UEDVT.

## Case presentation

A 51-year-old previously well Chinese gentleman presented with 1 day of progressive upper limb swelling and tightness. He did not report any personal history or family history of venous thromboembolic events. He worked as a flight steward who had flown from France to Singapore about 2 weeks prior to presentation. The day before symptom onset, he was working out in the gymnasium, where he completed his usual weight training and experienced no acute pain during the session. He had not sustained any trauma or underwent any surgical intervention to his arm prior to presentation. He is a non-smoker with no personal or family history of blood dyscrasias or malignancy.

**Fig. 1:**
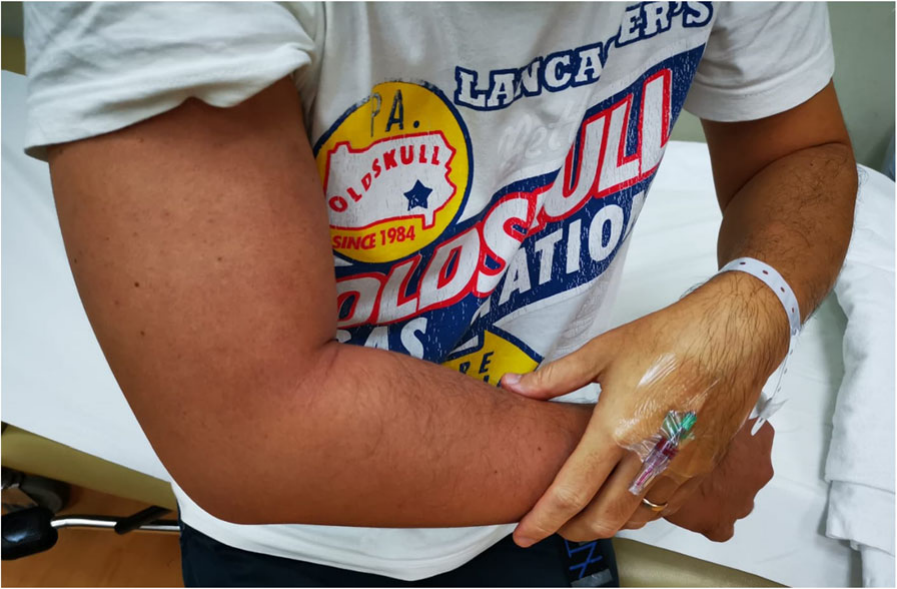
Right upper extremity oedema and hyperaemia

On presentation, he did not complain of any chest pain, breathlessness, or pleurisy. He was afebrile, with a blood pressure of 138/67 mmHg, heart rate 91 beats per minute, respiratory rate 19 breaths per minute, and oxygen saturation 98% on room air. On examination, he had a swollen, hyperaemic, and slightly oedematous right upper limb but without any tenderness or increased warmth (Fig. 1). Distal brachial and radial pulses remained strong. His left upper limb and both lower limbs were unremarkable.

**Fig. 2:**
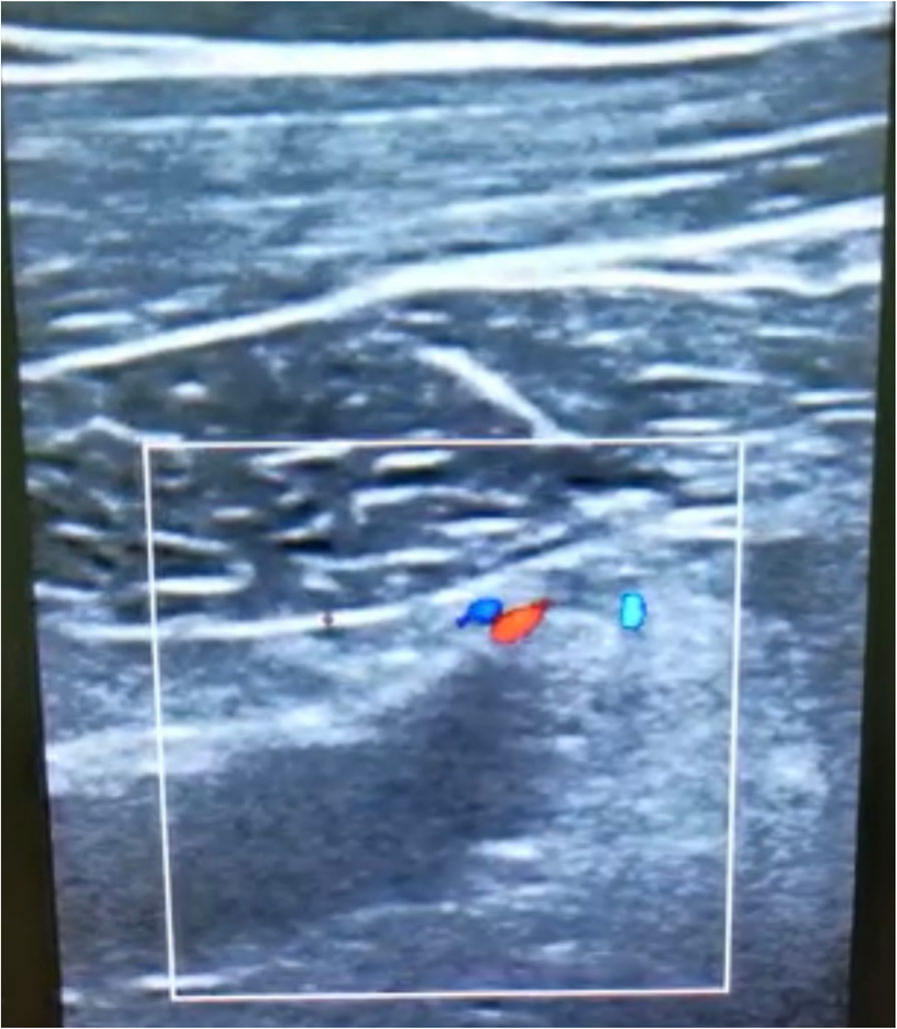
Long axis view of emergency department ultrasonography demonstrating clot in right subclavian vein

**Fig. 3:**
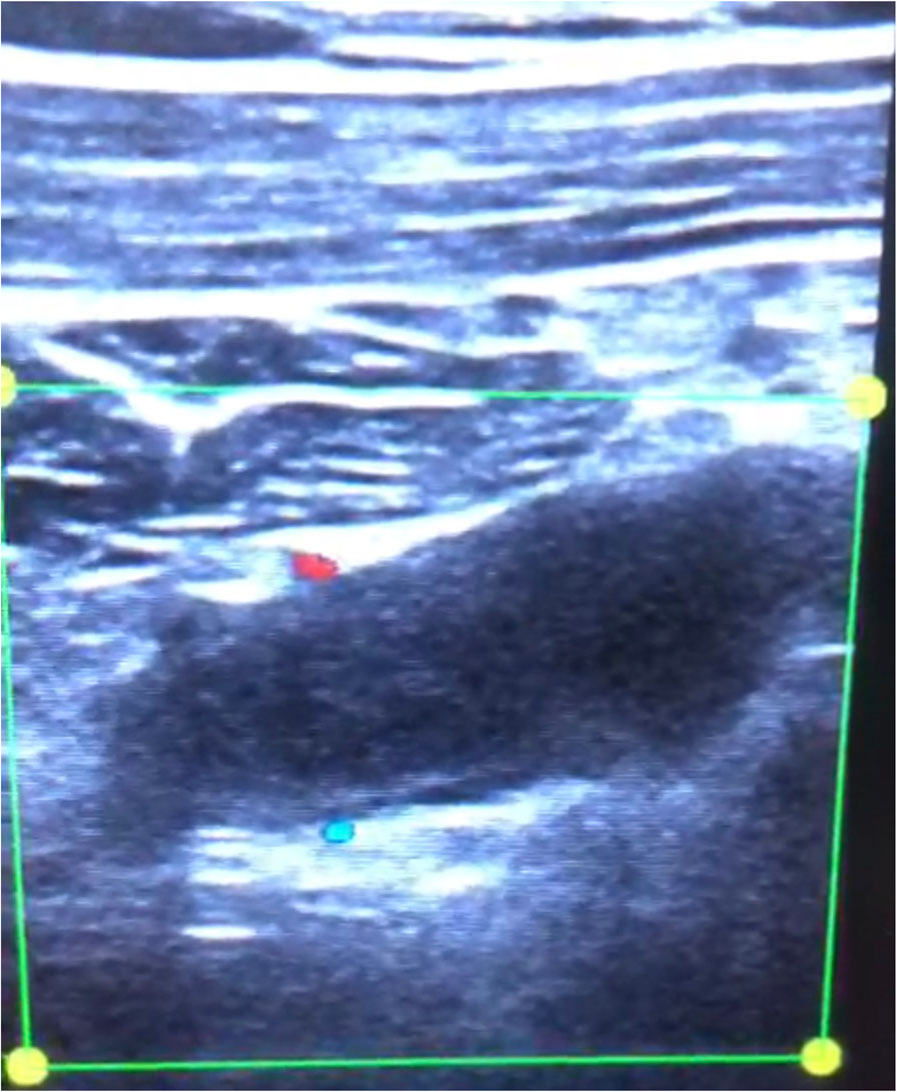
Longitudinal view of emergency ultrasound of right subclavian vein without augmentation

**Fig. 4:**
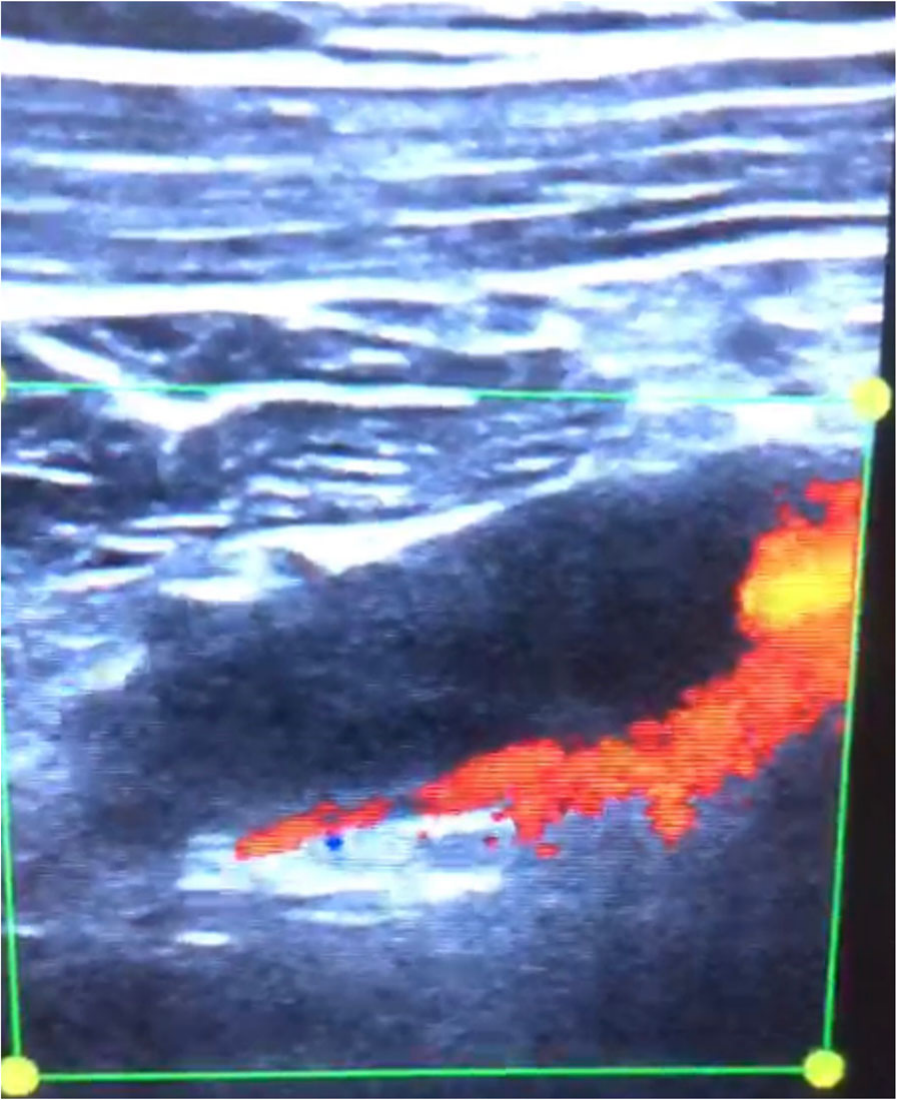
Longitudinal view of emergency ultrasound of right subclavian vein with augmentation

POCUS of the right upper limb was performed by the emergency physician with the patient in the supine position, and the brachial, axillary, and subclavian veins were carefully examined. The brachial vein was examined in the medial bicipital groove, the axillary vein as it courses in the apex of the axilla, and the subclavian vein in the infraclavicular fossa (beneath the outer and medial thirds of the clavicle) and the supraclavicular fossa [3]. This revealed a significant hypoechoic filling defect in the right subclavian vein that was highly suspicious for a large thrombus (Fig. 2). This finding was confirmed by abnormal single-point augmentation by arm squeeze manoeuvre to achieve venous compression distal to the site of examination with the ultrasound probe, where Fig. 3 demonstrates the Subclavian vein prior to the manoeuvre, and performing the manoeuvre did not result in a prompt increase in the volume of the venous signal as would be expected if the upper limb venous system was patent (Fig. 4).

Following discussion with vascular surgery, a computed tomography venogram was performed which confirmed the presence of a non-enhancing filling defect in the right subclavian vein as it passed between the clavicle and first rib, suggestive of extensive thrombi. Anticoagulation with enoxaparin was commenced and thrombectomy was scheduled for whilst the patient was in the emergency department. Interventional radiology subsequently performed a venography—which demonstrated acute thrombosis in the axillary and subclavian veins extending to the brachiocephalic junction—followed by retro-grade pulse spray thrombolysis with alteplase with mechanical thrombectomy. Magnetic resonance imaging studies revealed that the aetiology of his thoracic outlet syndrome was occlusion of the subclavian vein at the interval between the clavicle and first rib provoked by arm abduction. The patient eventually underwent excision of his right first rib to reduce the likelihood of recurrence of UEDVT.

## Discussion

Primary upper limb extremity deep vein thrombosis, also known as “Paget-Schroetter syndrome”, is thought to be a form of effort thrombosis. Repetitive strenuous activity causes mechanical compression by the clavicle, first rib, or hypertrophied shoulder muscles on the upper extremity vessel intima, leading to microtrauma and thrombosis [4, 5]. Secondary UEDVT is more common than primary UEDVT and is increasing in incidence due to the more prevalent use of mechanical indwelling devices such as central venous catheters, tunnelled central access lines, and pacing wires [6, 7].

There is currently no well-validated and standardized POCUS protocol for the diagnosis of UEDVT in the emergency department [8]. Unlike the two-point compression POCUS technique for lower extremity deep vein thrombosis (DVT), the subclavian vein is non-compressible and requires a different approach for assessment of presence of DVT. In addition, whilst an echogenic clot can be easily visualized, some hyperacute DVTs that have yet to become organized may appear completely hypoechoic. In these cases, abnormal colour Doppler flow could be the only tip-off to the presence of a hypoechoic clot [8].

Diagnostic accuracy can be further enhanced by performing distal venous compression to achieve augmentation. Augmentation was first described in the assessment of lower extremity DVT, where an abnormal augmentation response to venous compression by calf squeeze can aid in the detection of proximal lower extremity DVT [9]. McQueen et al. went on further to state that if compression POCUS examination is not possible, single-point augmentation can improve diagnostic accuracy for proximal lower limb DVT [9]. Augmentation via venous compression for upper limb venous system evaluation was also described in a case series by Bernier et al. This case report highlights how the arm squeeze manoeuvre to demonstrate abnormal augmentation can be performed in conjunction with colour Doppler assessment to assess the patency of the non-compressible subclavian vein as part of a bedside POCUS protocol.

## Conclusion

This case study highlights how rapid diagnosis of upper extremity DVT in the emergency department can be achieved by using a dedicated POCUS protocol (colour Doppler assessment followed by single-point augmentation manoeuvres) to evaluate the patency of the upper extremity venous system. This relatively unconventional technique can prove invaluable in facilitating expedited treatment of such a rare but dangerous and easily missed condition.

## Data Availability

Not applicable

## Abbreviations

UEDVT: Upper extremity deep vein thrombosis
POCUS: Point-of-care ultrasound
DVT: Deep vein thrombosis
